# Adipocytes protect fibroblasts from radiation-induced damage by adiponectin secretion

**DOI:** 10.1038/s41598-020-69352-w

**Published:** 2020-07-28

**Authors:** Elizabeth A. Kosmacek, Rebecca E. Oberley-Deegan

**Affiliations:** Department of Biochemistry and Molecular Biology, University of Nebraska Medical Center, 6.12.392 FPBCC, 985870 Nebraska Medical Center, Omaha, NE 68108 USA

**Keywords:** Cancer therapy, Radiotherapy, Extracellular signalling molecules

## Abstract

Prostate and colon cancers are among the most common cancers diagnosed annually, and both often require treatment with radiation therapy. Advancement in radiation delivery techniques has led to highly accurate targeting of tumor and sparing of normal tissue; however, in the pelvic region it is anatomically difficult to avoid off-target radiation exposure to other organs. Chronically the effects of normal urogenital tissue exposure can lead to urinary frequency, urinary incontinence, proctitis, and erectile dysfunction. Most of these symptoms are caused by radiation-induced fibrosis and reduce the quality of life for cancer survivors. We have observed in animal models that the severity of radiation-induced fibrosis in normal tissue correlates to damaged fat reservoirs in the pelvic region. We hypothesize that adipocytes may secrete a factor that prevents the induction of radiation-associated fibrosis in normal tissues. In these studies we show that the adipokine, adiponectin, is secreted by primary mouse adipocytes and protects fibroblasts from radiation-induced cell death, myofibroblast formation, and senescence. Further, we demonstrated that adiponectin does not protect colorectal or prostate cancer cells from radiation-induced death. Thus, we propose that adiponectin, or its downstream pathway, would provide a novel target for adjuvant therapy when treating pelvic cancers with radiation therapy.

## Introduction

Radiation is used to treat a variety of pelvic cancers, including prostate and colorectal cancers. While radiation is an effective tool to treat these cancers, radiation also damages surrounding normal tissues. The chronic side effects of radiation treatment are similar for either prostate or colorectal cancer and include: urinary frequency, urinary incontinence, proctitis, erectile dysfunction, and bowel fibrosis^1–4^. Most of these long-term side-effects are due to radiation-induced fibrosis.

Fibroblasts are the cell type that is most studied in the context of radiation-induced fibrosis. In particular, fibroblasts, which function to maintain normal extracellular matrix (ECM) by producing proteins such as collagen and fibronectin, can become chronically activated after radiation exposure^5^. Activated fibroblasts, or myofibroblasts, are initiated by ionizing radiation that activates the TGFβ pathway and chronic production of reactive oxygen species (ROS), which ultimately produces aberrant amounts of ECM^6–8^. Irradiation of fibroblasts in normal tissue can lead to cell death, fibroblast activation, or cellular senescence, all of which can produce long term side effects including inflammation, fibrosis, and chronic tissue dysfunction^5,9^.

There are other cell types involved in fibrosis besides fibroblasts. Immune cells can provide a pro-fibrotic niche to activate fibroblasts and cause epithelial to mesenchymal transformation^10–12^. Additionally, the pelvic region contains a large depot of visceral and subcutaneous adipose tissue. The role of adipocytes in radiation-induced fibrosis is unknown. Previously we have published that in mice, pelvic irradiation damages gonadal fat tissues resulting in total tissue atrophy, reduction in adipocyte size, and increased inflammatory cell infiltration^13^. We also observed that animals whom experienced significantly less acute and chronic normal tissue damage in both skin and bladder correlated to less damage in the local fat tissue. Fat is a dynamic organ capable of systemically regulating body metabolism and exerting anti-inflammatory, antifibrotic, and antioxidant effects^14^. We hypothesized that healthy fat may play a protective role against radiation-induced fibrosis by reducing cell death, fibroblast transformation, and cellular senescence.

Adipocytes secrete many factors that regulate cell and tissue function systemically^15,16^. One of these factors is adiponectin. In a variety of disease models, adiponectin has been demonstrated to inhibit inflammation, ROS, and fibrosis though the upregulation of AMP-activated protein kinase (AMPK)^17–23^. Adiponectin binds directly to transmembrane receptors, AdipoR1, AdipoR2 or T-cadherin, to affect intracellular signaling^24,25^. In skin and lung fibroblasts, AdipoR1 seems to be the receptor most important in mitigating fibrotic signaling in these cells^22,26,27^. However, in the liver, AdipoR2 appears to be the preferential target of APN to reduce fibrosis^28,29^. T-cadherin is mainly expressed in endothelial cells and neuronal tissues and has not been well characterized in the context of adiponectin mediated fibrosis. There have been two studies demonstrating that 12 months after exposure to sub-lethal doses of whole body irradiation (WBI), AdipoR1 and AdipoR2 protein levels were significantly reduced in the colon; however, serum levels of adiponectin were not affected^30,31^. These data indicate that radiation may have chronic effects on the adiponectin signaling pathway.

In the present study, we show that primary mouse adipocytes secrete the protective adipokine, adiponectin. Several studies have determined that adiponectin can modulate ROS and suggest its therapeutic role in ROS driven pathologies. Further, it has been demonstrated in both histological samples and in normal fibroblast cell lines that prostate fibroblasts express the AdipoR1 and AdipoR2 receptors^32,33^. However, it has never been shown that adiponectin can confer protection from radiation-induced damage to normal fibroblast cells. We show that both adipocytes and exogenous adiponectin protect primary fibroblasts from radiation-induced cell death, myofibroblast transformation, and cellular senescence. Notably, adiponectin does not protect cancer cells from radiation killing. The data shown herein suggest a role for the therapeutic use of adiponectin during radiation cancer therapy to prevent radiation-induced fibrosis of normal tissues and improve the quality of life for cancer survivors.

## Results

### Co-culture with adipocytes protects fibroblasts from radiation-induced cell death

**Figure 1:**
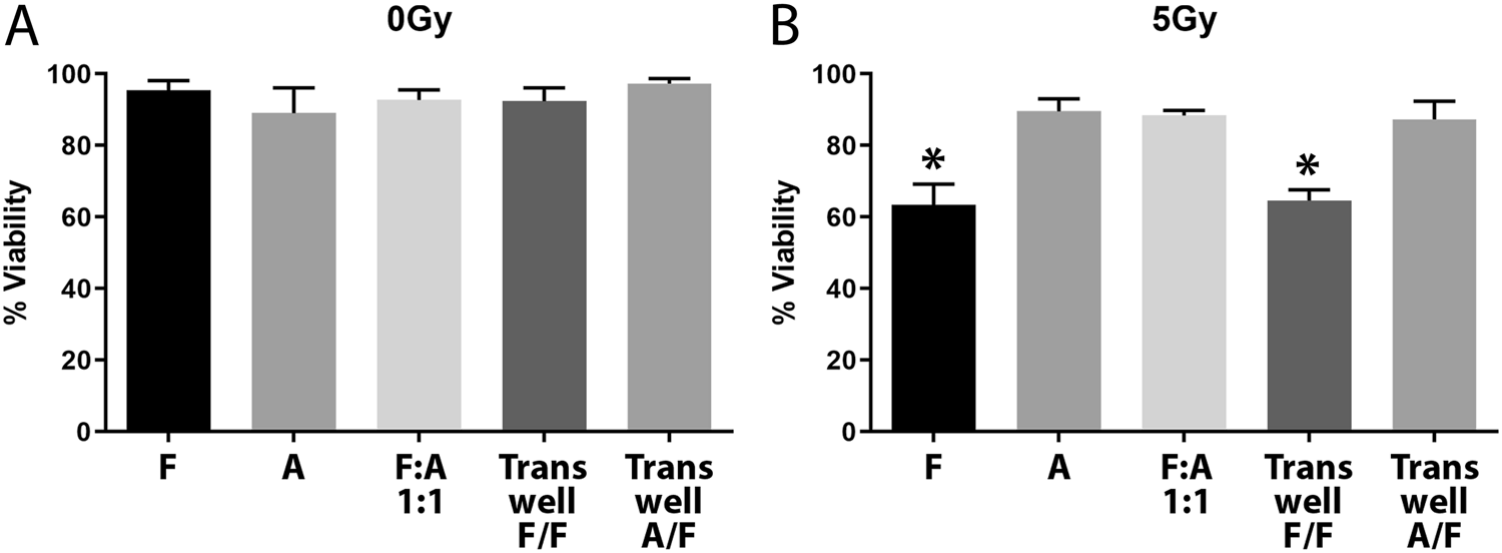
Co-culture with adipocytes enhances fibroblast viability following radiation. Cell cultures were either (**A**) sham irradiated or treated with (**B**) 5 Gy of X-rays then assayed for viability with trypan blue. The various culture conditions included fibroblasts (F), adipocytes (A), a 1:1 mixture of fibroblasts and adipocytes (F:A), or fibroblasts with transwell inserts containing fibroblasts (F/F) or adipocytes (A/F). A 1-way ANOVA with post hoc Tukey test was used to calculate significance (*p* < 0.0001, n = 3) between groups, (*) indicates significantly lower viability as compared to sham conditions.

Primary adipocytes from gonadal fat pads and fibroblasts from the prostates of C57BL/6 mice were isolated and cultured for 2 weeks. Adipocytes and fibroblasts were grown alone or co-cultured at a 1:1 ratio for 24 h and then exposed to 5 Gy of radiation. After 5 days incubation, the cell viability was analyzed by trypan blue staining. Fibroblasts alone had a significant (*p* < 0.0001) 40% reduction in cell viability following radiation exposure, while adipocyte viability was unchanged following radiation (Fig. 1A, B). When fibroblasts were co-cultured with adipocytes, the fibroblasts were protected from radiation-induced cell death (Fig. 1A, B). To further characterize how adipocytes were protecting fibroblasts, we utilized a transwell system to co-culture the cells without the benefit of cell-to-cell contact. Fibroblasts were seeded on the bottom of a well and then fibroblasts or adipocytes were seeded on a transwell insert. The transwell was removed during radiation treatment to maintain the health of those cells. We had already determined that this dose of radiation was not damaging to adipocytes alone or when mixed with fibroblasts. However, we did not want to damage fibroblasts in the transwell, and for consistency all transwells were removed during radiation treatment. Additionally, the transwell was removed to prevent artifacts possibly introduced by a physical barrier between the radiation source and the fibroblasts on the culture surface. When fibroblasts were grown in the transwell, no protection was conferred to the irradiated fibroblasts in the culture dish. However, when adipocytes were grown in the transwell, viability of the irradiated fibroblasts was substantially protected (Fig. 1A, B). These co-culture experiments demonstrate not only that adipocytes confer protection to fibroblasts from radiation but that cell–cell contact is not required for this protection. Thus, indicating that a secreted factor from the adipocytes is likely the source of the protection.

### Conditioned media from adipocytes protects fibroblasts from radiation-induced cell death

**Figure 2:**
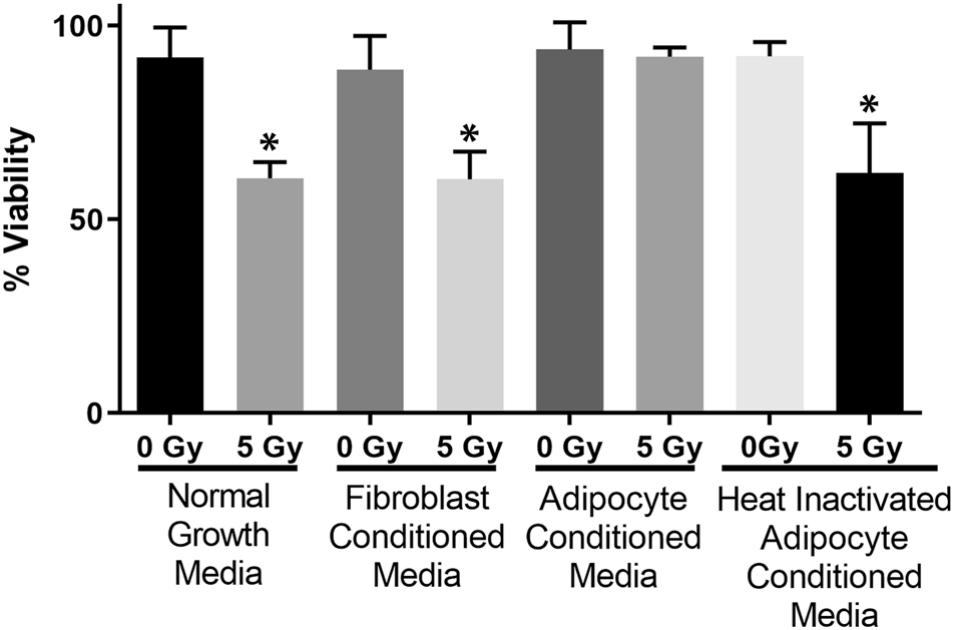
Conditioned media from adipocytes enhances fibroblast viability following irradiation. Fibroblasts were incubated in either normal growth media, fibroblast conditioned media, or adipocyte conditioned media with and without heat inactivation. Statistical significance was assessed by a 1-way ANOVA with post hoc Tukey’s test for multiple comparisons. (*) Indicates viability was significantly reduced at 5 days following 5 Gy irradiation (*p* < 0.01, n = 3) as compared to sham irradiated controls.

To further demonstrate that a secreted factor from adipocytes confers protection to fibroblasts from radiation-induced cell death, conditioned media was collected from cultured adipocytes. Fibroblasts were seeded in 6 well plates overnight and one hour prior to radiation exposure, normal growth media was replaced with fresh media or conditioned media from either adipocytes or fibroblasts. Cultures were exposed to 5 Gy of radiation, cultured for 5 days, and then assayed for cell death with trypan blue. As in the co-culture experiments, cells in normal growth media or fibroblast conditioned media had reduced viability by 40% (*p* = 0.0018), but adipocyte conditioned media provided protection equivalent to sham irradiated cultures (Fig. 2). To better understand the biochemical properties of the secreted factor, the adipocyte media was heat inactivated prior to introduction to the fibroblast culture. Heat inactivation destroyed the protective capabilities of the media (*p* = 0.0026) (Fig. 2). This indicated that the secreted factor was likely a protein.

**Figure 3:**
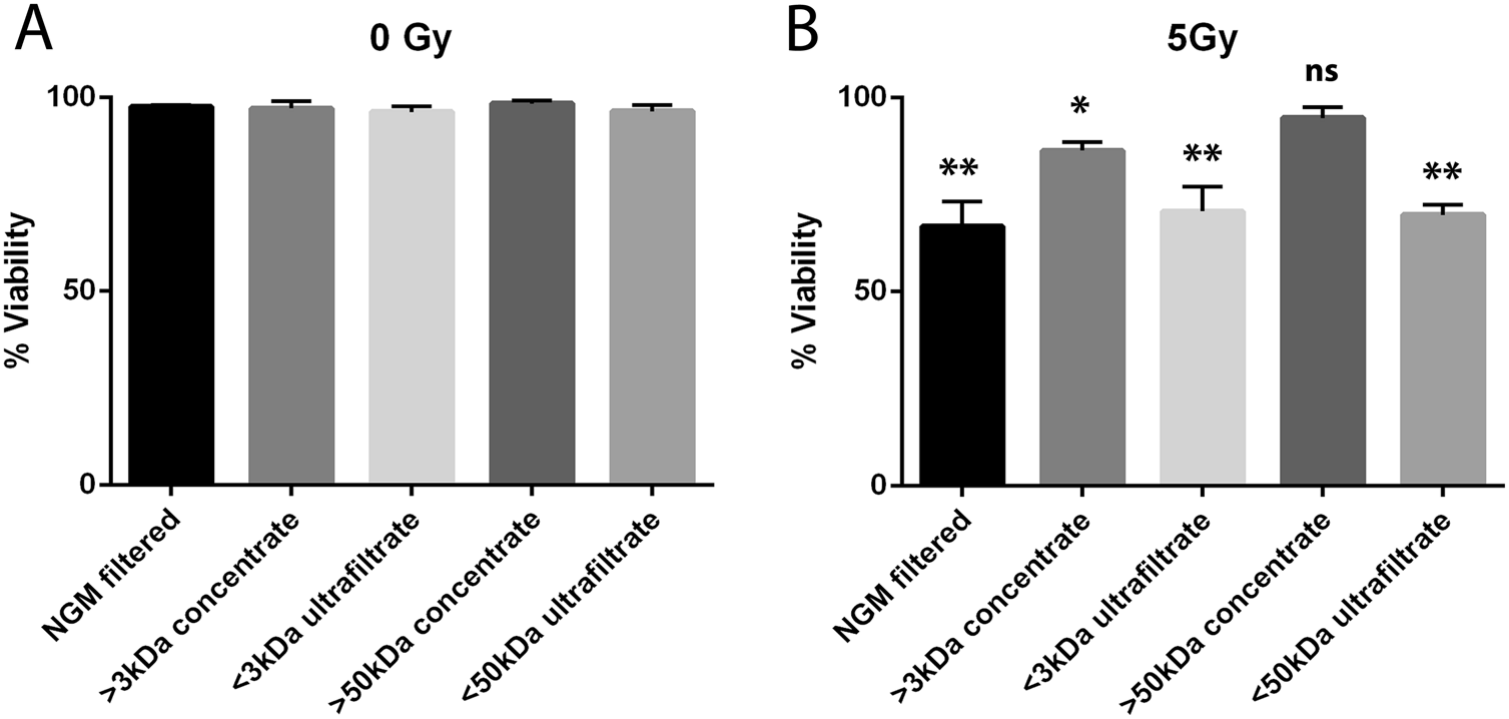
A protein of > 50 kDa is responsible for enhanced fibroblasts viability following irradiation. Size exclusion columns were used to concentrate proteins either at > 3 kDa or > 50 kDa. We also tested the ultrafiltrates that contain protein < 3 kDa and < 50 kDa in normal concentrations. The normal growth media (NGM) and ultrafiltrates had significantly reduced viability 5 days after 5 Gy radiation treatment. The > 3 kDa concentrate provided some enhancement to viability but was still significantly reduced, whereas the > 50 kDa concentrate protected fully from radiation-induced cell death. Statistical significance was determined using a 1-way ANOVA with post hoc Tukey’s test for multiple comparisons (*) indicates *p* < 0.05 and (**) indicates *p* < 0.001 as compared to all sham irradiated conditions. n = 3 for all experimental conditions.

Size exclusion columns were used to separate media containing varying sizes of proteins to aid in identifying the possible protective protein secreted by adipocytes. Columns that separate proteins of 3 kDa and 50 kDa were used to isolate four versions of conditioned media; > 3 kDa concentrate, < 3 kDa ultrafiltrate, > 50 kDa concentrate, and < 50 kDa ultrafiltrate. Each of these medias was applied to fibroblasts in culture one hour prior to either sham or 5 Gy radiation treatment, and then cultured further for 5 days and assayed for cell viability. Cell viability was not affected by the media filtration alone as measured in the sham cultures (Fig. 3A). As with cells cultured in normal growth media, the medias containing proteins < 3 kDa or < 50 kDa had 40% reduced viability following 5 Gy irradiation (*p* < 0.0001) (Fig. 3B). Media containing > 3 kDa protein was moderately protective but still had a significant (*p* = 0.0478) reduction in viability of about 20% (Fig. 3B). Media containing only proteins > 50 kDa protected fibroblasts similar to control levels (Fig. 3B). From these data we determined a protein > 50 kDa was the most likely candidate being secreted from adipocytes and protecting fibroblasts from radiation damage.

### Adiponectin is secreted from adipocytes and protects fibroblasts from radiation-induced cell death

**Figure 4:**
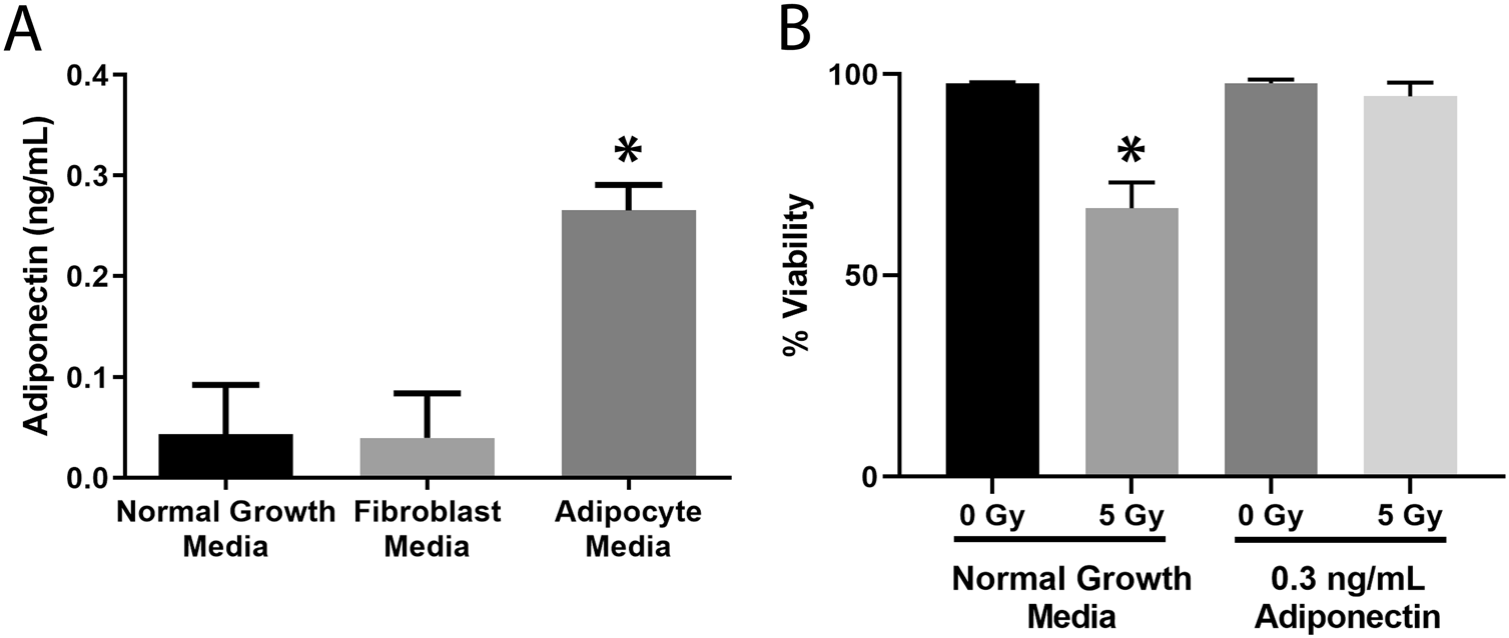
Adiponectin is a secreted factor present in conditioned media and exogenous addition of recombinant adiponectin protects fibroblasts from radiation-induced cell death. (**A**) Normal growth media, fibroblast conditioned media, and adipocyte conditioned media were assayed with an ELISA for mouse adiponectin. Adipocyte media contained significantly more (*p = 0.001) adiponectin than the other medias tested. (**B**) Recombinant mouse adiponectin equivalent to concentrations found in adipocyte conditioned media, 0.3 ng/mL, was added to normal growth media prior to 5 Gy irradiation. Cells were assessed for viability 5 days later using trypan blue. (*) indicates significantly less viability than all other groups (*p* < 0.0001). All statistical significance was determined by a 1-way ANOVA followed by post hoc Tukey’s test for multiple comparisons, n = 3 for all experiments.

After surveying the literature, we identified some large proteins that are secreted by adipocytes which also have cytoprotective effects, one of these proteins was adiponectin. Adiponectin is a 30 kDa hormone secreted specifically by adipocytes and it exists in trimer (90 kDa), hexamer (180 kDa), and 12–18 mer (> 300 kDa) in its native conformations^34^. Using a mouse adiponectin ELISA, the concentration of secreted adiponectin in adipocyte media was detected at 0.3 ng/mL (Fig. 4A). The normal growth media and fibroblast conditioned media were also assayed for adiponectin, and the detected levels were near zero.

To determine whether adiponectin protects cell viability following radiation of fibroblasts, normal growth media was spiked with 0.3 ng/mL recombinant mouse adiponectin one hour prior to radiation and viability was assessed 5 days later. Exogenous addition of adiponectin was able to prevent radiation-induced cell death as effectively as adipocyte conditioned media (Figs. 2, 4B).

### Adipocyte media and adiponectin prevent radiation-induced fibroblast activation

**Figure 5:**
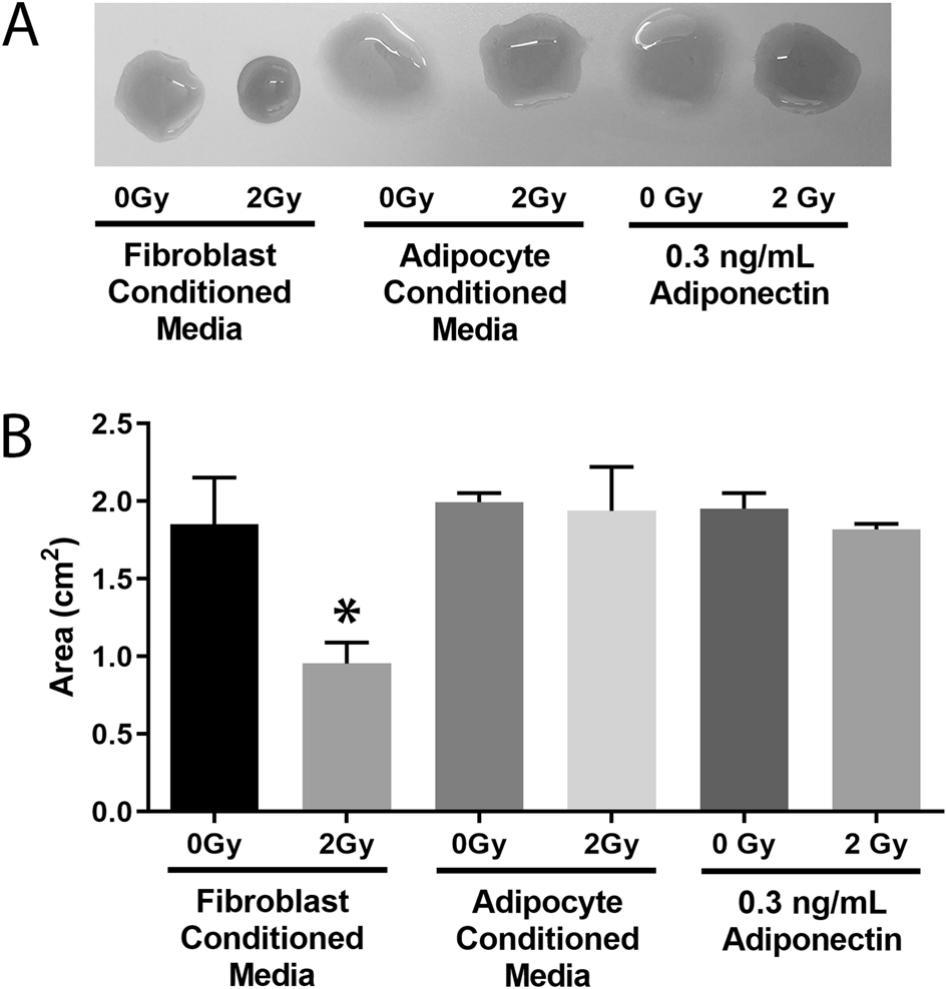
Adipocyte conditioned media and exogenous adiponectin protect fibroblasts from radiation-induced activation. Fibroblasts were irradiated with 2 Gy in the presence of either fibroblast conditioned media, adipocyte conditioned media, or exogenous adiponectin. Two days later the cells were embedded in rat tail collagen discs and observed for their ability to contract extracellular matrix. (**A**) Five hours after embedding fibroblasts in collagen, the discs were removed from the incubator and imaged. (**B**) ImageJ was used to measure the total area of the contracted discs. For each biological replicate, three technical replicates were performed. Statistical analysis was assessed by a 1-way ANOVA followed by post hoc Tukey’s test for multiple comparisons. (*) indicates significant increase (*p* < 0.0001) as compared to any sham irradiated control. All experiments were completed in triplicate with three technical replicates each.

A normal fibroblast expresses very little α-smooth muscle actin (α-SMA) and, therefore, has no contractility properties. One attribute of an activated fibroblast is the ability to contract its extracellular matrix. Fibroblasts were embedded in rat tail collagen 2 days after 2 Gy of irradiation, the collagen discs were allowed to contract for approximately 5 h then the area of the disc was measured with ImageJ. As expected, fibroblasts exposed to irradiation alone significantly (*p* = 0.0008) contracted the collagen disc, indicating that these fibroblasts were activated (Fig. 5B). In contrast, when fibroblasts were irradiated in the presence of adipocyte conditioned media or exogenous adiponectin the discs did not significantly contract (Fig. 5A, B).

### Radiation-induced senescence is reduced by adipocyte media and exogenous adiponectin

**Figure 6:**
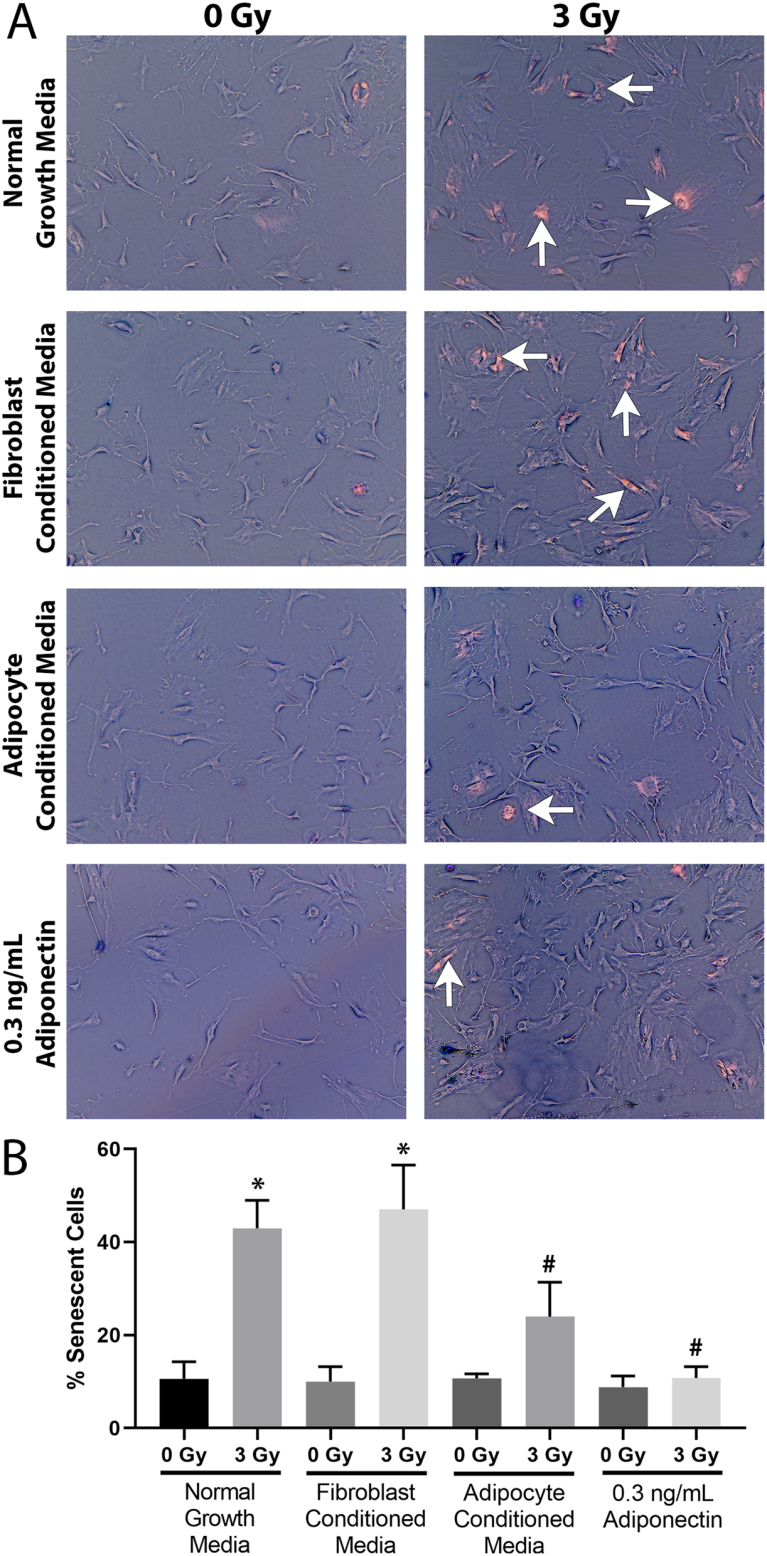
Adipocyte conditioned media and exogenous adiponectin protect fibroblasts from radiation-induced senescence. Fibroblasts were irradiated with 3 Gy in the presence of either normal growth media, fibroblast conditioned media, adipocyte conditioned media, or exogenous adiponectin. Three days later the cultures were assayed for β-galactosidase activity. (**A**) Representative images of cells stained for β-galactosidase activity, images were inverted to aid in visualizing positively stained cells (examples indicated by white arrows), which appear bright pink. (**B**) Graphical representation of quantified images as percent positively staining cells. Statistical analysis was determined by a 1-way ANOVA followed by post hoc Tukey’s test for multiple comparisons, n = 3 for all conditions. (*) indicates significant increase (*p* < 0.0001) as compared to any sham irradiated condition. (#) indicates significantly less senescence (*p* < 0.001) as compared to either irradiated fibroblasts in normal growth media or fibroblast conditioned media.

Radiation can induce cellular senescence, which contributes to the further activation of neighboring fibroblasts and promotes fibrosis. Fibroblasts were exposed to 3 Gy of radiation and then assayed for β-galactosidase activity 3 days post-radiation treatment. Fibroblast cultures grown in normal growth media or grown in fibroblast conditioned media had 30% more senescent cells as compared to their unirradiated controls (*p* < 0.0001) (Fig. 6A, B). However, when cultured in adipocyte conditioned media or with exogenous adiponectin added to normal growth media, an insignificant increase in senescent cells (10% and 0%, *p* = 0.1140 and *p* = 0.9998 respectively as compared to unirradiated controls) were observed (Fig. 6A, B).

### Adiponectin does not protect cancer cells from radiation-induced growth inhibition

**Figure 7:**
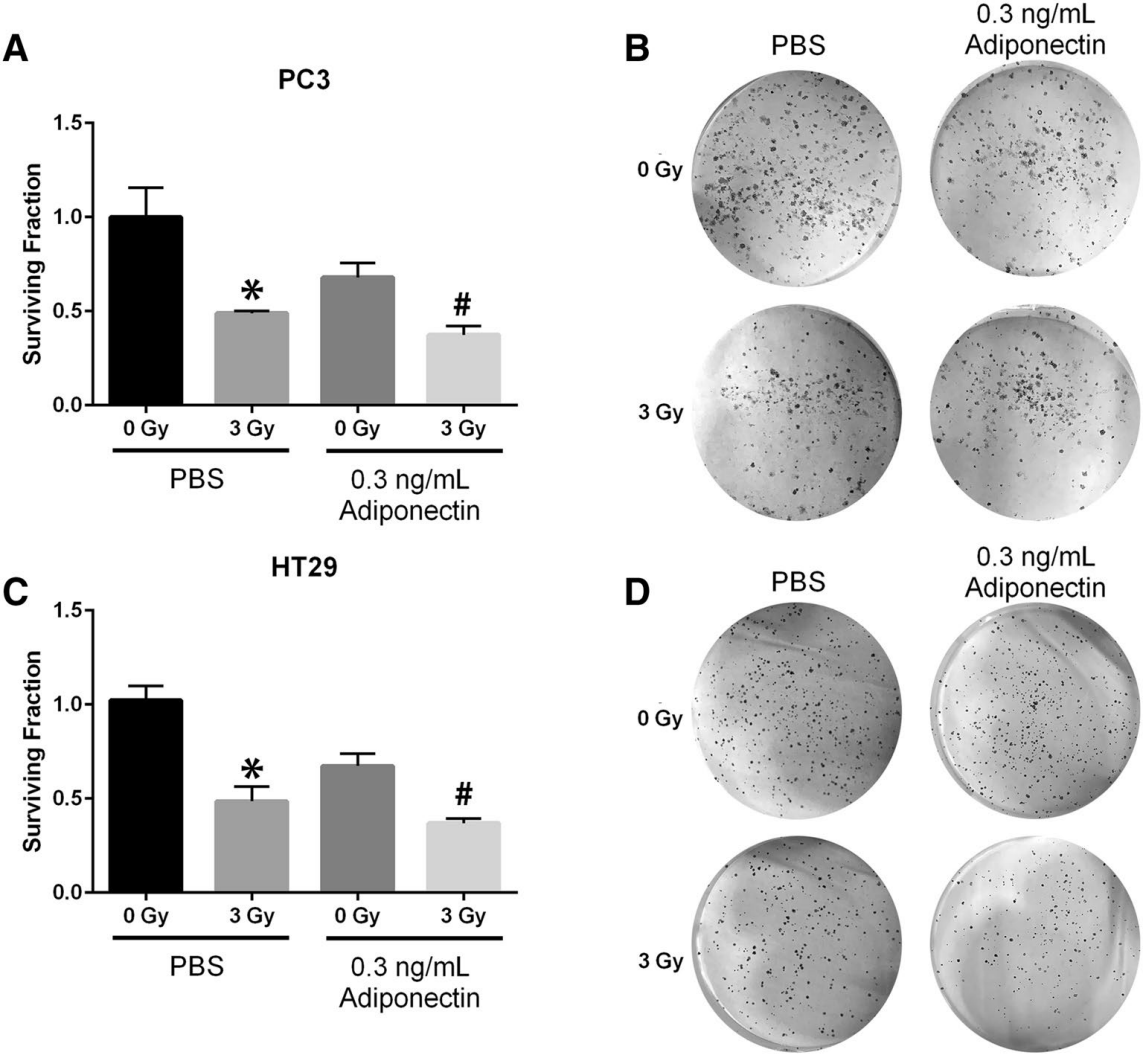
Adiponectin alone can reduce cancer cell growth and does not protect cancer cells from radiation damage. (**A**, **B**) Prostate cancer cells, PC3, and (**C**, **D**) colorectal cancer cells, HT29, were treated with PBS or 0.3 ng/mL of human recombinant adiponectin then either sham irradiated or treated with 3 Gy X-rays. Cells were immediately detached and seeded at low density for 10 days then fixed, stained, and colonies enumerated to determine clonogenic survival, quantified in (**A**, **C**). Representative images of stained colonies are displayed in (**B**, **D**). For each biological replicate (n = 3), three technical replicates were performed. Statistical analysis was performed with a 1-way ANOVA followed by post hoc Tukey’s test for multiple comparisons. For both cell lines adiponectin showed statistically significant differences between paired sham and irradiated treatments where (*) indicates *p* < 0.001 and (#) indicates *p* < 0.05.

If adiponectin is to be considered as a viable radioprotector to be used during cancer radiotherapy, it is imperative that adiponectin does not protect cancer cells from radiation damage. Prostate and colorectal cancer cell lines were either sham irradiated or given 3 Gy of X-rays in the presence of PBS or 0.3 ng/mL of recombinant adiponectin, and assayed for clonogenic survival. Prostate cancer (PC3) clonogenicity was significantly reduced by either adiponectin treatment (*p* = 0.0104) or radiation treatment (*p* = 0.0006) (Fig. 7A, B). For combined treatment of radiation and adiponectin, a further reduction in clonogenic survival as compared to cell cultures receiving only radiation treatment was observed (*p* = 0.0137). Colorectal cancer (HT29) also showed significant reduction in clonogenicity after 3 Gy of radiation exposure (*p* < 0.0001). As with PC3, the HT29 clonogenicity was reduced by adiponectin alone (*p* = 0.0007), and for combined therapy with adiponectin there was an additive effect to the radiation treatment (*p* = 0.0018) (Fig. 7C, D). Thus, adiponectin does not protect cancer cells from radiation damage and, in fact, imparts a further reduction in clonogenic survival when applied as an adjuvant therapy.

## Discussion

Prostate and colorectal cancers are often treated with pelvic radiation therapy and while many measures are taken to spare normal tissue from radiation exposure there is often some exposure to surrounding tissues that can lead to radiation-induced chronic side-effects. Currently there is no cure for radiation-induced fibrosis. It is still largely unknown why certain individuals develop radiation-induced fibrosis while others have almost no complications. We and others have shown that radiation can induce fibroblasts to differentiate into myofibroblasts, an activated form with highly contractile properties and that produce and deposit collagen into the extracellular matrix^8,13,35,36^. When these cells remain activated and unchecked the excess collagen accumulates over time which, eventually results in fibrosis and organ dysfunction. However, there are other cell types besides fibroblasts that are exposed to irradiation during external beam therapy for pelvic cancers that could be involved in fibrosis as well.

One cell type we speculate may be playing a role in the fibrotic process is the adipocyte. Although adipocytes are present in most organs and are present in the greater omentum of the pelvis, adipose tissue has not been well studied in the context of radiation exposure. What little has been reported demonstrates that adipose tissue is radiosensitive and prone to developing inflammation^37^. In addition, clinical studies have demonstrated that cancer patients with metabolic disorders are more prone to radiation-induced toxicities^38–41^. We have previously shown that, in animals with radiation-induced fibrosis caused by pelvic irradiation, the pelvic fat pads are atrophied as well as highly inflamed. In contrast, in animals with no radiation-induced fibrosis, the pelvic fat pads are intact with no inflammation^13^. Thus, the purpose of this study was to better understand the role of healthy adipocytes in the prevention of radiation-induced injury.

In this study we have shown that co-culturing fibroblasts with adipocytes protects fibroblasts from cell death after radiation treatment. Further, this protection is also observed when culturing fibroblasts in adipocyte conditioned media. This means that adipocytes do not require cell-to-cell contact to protect fibroblasts, and that adipocytes are secreting a protective factor. Adipocytes secrete signaling molecules and proteins known as adipokines, which often have anti-inflammatory, anti-fibrotic, and anti-apoptotic functions. To determine if the adipocyte secreted factor was a protein, we heat-inactivated adipocyte conditioned media. We found that the protection was ablated after heat inactivation; thus, a secreted protein was responsible for protecting fibroblasts from radiation-induced cell death.

There are several decades of research devoted to identifying various adipokines secreted from adipocytes and their physiological roles. To narrow down the possible protective factor in our study, we used size exclusion centrifuge filters to separate proteins of varying molecular weight. By repeating our viability assay in these filtered medias, we were able to confirm that a protein of > 50 kDa was the major protective factor in our system. We would have expected the > 3 kDa and > 50 kDa concentrates to protect equally as both should have contained the same large proteins. We suspect that another inhibitory protein may have been concentrated by the > 3 kDa column, masking the protective effects of adiponectin. While adiponectin has not been rigorously described in the context of radiation, several papers have described the anti-fibrotic properties of adiponectin, which led us to hypothesize that adiponectin was the likely candidate. We measured the concentration of adiponectin in our adipocyte conditioned media and found it to be 0.3 ng/mL. This concentration of adiponectin is similar to other concentrations reported from in vitro studies using cultured adipocytes^42^. However, levels of adiponectin are greater in the blood serum and are in the range of 3–15 µg/mL.

In agreement that adiponectin is likely a major factor in protecting fibroblasts from irradiation damage, when adiponectin was exogenously added we observed complete evasion of radiation-induced cell death. In addition, conditioned adipocyte media or exogenous adiponectin inhibited the activation of fibroblasts. Indicating that adiponectin was likely the factor in reducing the transformation of a fibroblast to a myofibroblast after radiation exposure. This data is also important because it points to the role that healthy adipocytes and adiponectin may have in reducing radiation-induced fibrosis.

Another component of the fibrotic process is the production of senescent cells, which propagate the proinflammatory signaling that drives fibroblast transformation. We measured the induction of senescent cells using a β-galactosidase activity assay and found that adipocyte conditioned media and recombinant adiponectin both reduced the number of senescent cells at 3 days post-radiation exposure. As the total cell number was visibly greater in adiponectin treatment it is uncertain if senescence was prevented, or if normal cell growth may have masked the senescent cell population. However, we know that the reduction of senescent cells was not due to more cell death as cell death was not visibly enhanced in the treated cell cultures, and further when higher doses of radiation were used, the adipocyte media or exogenous adiponectin prevented cell death in fibroblast cultures.

We used several doses of radiation throughout this study to elicit different responses from fibroblasts; 5 Gy to induce cell death, 3 Gy to promote senescence, and 2 Gy to activate myofibroblast transformation. When radiation therapy is delivered clinically the isodoses to surrounding normal tissues are minimized with image guidance and arc beam treatment planning. The amount of radiation delivered to normal tissue at maximal voltage may range up to 20% of the dose delivered to the tumor^43^. Thus, it is reasonable to assume cells in surrounding tissues will see variable doses by several Gy over the course of fractionated radiation therapy and that those cells will respond differently according to those doses.

There has been only one study conducted to investigate the effect of adiponectin on radiation damage^44^. In this study, adiponectin knock out (APN^−/−^) or WT mice were treated with 3 or 6 Gy of whole body irradiation (WBI) and then intestinal tissues or bone marrow were analyzed at 1 and 5 days post-radiation. The blood cells from APN^−/−^ mice had significantly more microneuclei formation, indicating that the DNA was more damaged in the blood cells when adiponectin was removed. However, the overall gross damage was similar between WT and APN^−/−^ mice at these acute time points. Based on our data and other studies demonstrating that adiponectin is anti-fibrotic, we would surmise that if this study was carried out longer, then APN^−/−^ mice would have more fibrosis than the WT mice. This study also used large doses of WBI that also may have overwhelmed the protective effect of adiponectin. Our model represents a more clinical model of cancer radiotherapy, which adiponectin appears to protect from radiation-induced damage.

As we have shown that adiponectin is a strong radioprotector for normal fibroblast cell populations it is of concern that it may also protect cancer cells from radiation damage. It has been shown in patients with prostate or colorectal cancers, as well as in the particular cancer cell lines used in this study (PC3 and HT29), that these tumor cells express receptors for adiponectin^45–48^. There is a wealth of published research showing adiponectin may actually have anti-tumor properties. Several studies have shown in tumor cells that adiponectin can activate AMPK which inhibits the mTOR pathway suppressing VEGF-A and, therefore, neovascularization of tumors^48–50^. Additionally, a study with both prostate and colorectal cancer cell lines demonstrated that adiponectin signaling through AMPK reduced mTOR activation and consequently reduced protein synthesis which led to cell cycle arrest^51^. Particularly in models of obesity where adiponectin is known to be lower in obese individuals, adiponectin treatment promotes tumor cell death^38,52^. In addition, prostate and colorectal cancer patients often have reduced serum adiponectin levels and the addition of adiponectin reduces prostate and colorectal tumor growth in animal models^45,53–55^. However, there is no data demonstrating the role of adiponectin during radiation. We, therefore, investigated the clonogenic capacity of prostate and colorectal tumor cell cultures when irradiated in the presence of adiponectin. We found that adiponectin alone or in combination with radiation resulted in the reduction of cancer cell growth following treatment. Thus, this data provides the ground work to pursue the role of adiponectin as a radioprotector for use during cancer therapy.

While we did not conclusively show that adiponectin is the only protective factor secreted from adipocytes, we did show that adiponectin is produced by adipocytes and that protection from radiation-induced damage occurred when this protein was added exogenously. There could be other factors that are secreted from the adipocytes besides adiponectin that are also responsible for protection from radiation-induced damage. This will be the focus of future studies.

In conclusion, this is the first study to demonstrate that healthy adipocytes confer protection from radiation-induced damage of fibroblasts. It is also the first study to demonstrate that adiponectin could provide a unique adjuvant therapy to radiation in the context of cancer therapy. We acknowledge that there is difficulty in producing and administering recombinant adiponectin to patients clinically. However, the pathways downstream of the adiponectin receptors are very well studied in other model systems. Going forward we plan to tease these pathways out during radiotherapy and target these affected pathways. We believe this is a novel and promising approach to mitigate radiation damage to normal tissues that would greatly improve the quality of life for cancer survivors. Specifically, we predict cancer patients with metabolic disorders and obese patients with low adiponectin levels would benefit the most from this targeted therapy.

## Materials and methods

### Experimental animals

6–8 week old male C57BL/6J mice, obtained from Jackson Laboratories, were used in this study. The mice were housed at the University of Nebraska Medical Center (UNMC) and exposed to a 12 h light/12 h dark cycle and fed and watered ad libitum. This study was carried out in strict accordance with the recommendations of the Guide for the Care and Use of Laboratory Animals of the National Institutes of Health. All procedures were approved by the institutional animal care and use committee at UNMC (14-054-08-FC).

### Isolation and culture of mouse primary prostate fibroblasts

Mouse primary fibroblasts were isolated as previously described^8,35^. Prostates were isolated from 6–8 week old C57BL/6J mice. Tissue was roughly minced with a scalpel then digested in 5 mg/mL collagenase I (Thermo Fisher, cat. 17100017, Waltham, MA, USA) for 30 min at 37 °C. Liberated cells and tissue fragments were then cultured for 2–3 weeks in Dulbecco’s Minimal Essential Media (DMEM), supplemented with 10% FBS, 1% penicillin/streptomycin and 1% non-essential amino acids. Cultures were maintained at 5% CO_2_ and 37 °C. After 5 days of culture only fibroblasts were present. Purity of the cells was determined using ERTR7 (Abcam, cat. ab51824, Cambridge, MA, USA), a fibroblast marker and Keratin17 (Cell Signaling, cat. 4543, Danvers, MA, USA), an epithelial cell marker. All experiments were repeated in triplicate using primary fibroblasts cells collected from prostates of different mice.

### Isolation and culture of mouse primary adipocytes

Isolation of primary adipocytes was adopted from Poglio et al.^37^. Gonadal fat pads were isolated from 6–8-week-old C57BL/6 J mice and visible blood vessels or other tissue was carefully dissected out. The fat tissue was agitated in 2 mg/mL collagenase I at 37 °C for 45 min until the mixture became cloudy. The cells were pelleted by centrifugation at 400×*g* for 4 min, the lipid layer was discarded. The cells were cultured in DMEM, supplemented with 10% FBS, 1% penicillin/streptomycin and 1% non-essential amino acids in a humidified incubator at 37 °C and 5% CO_2_. Media was changed every other day to remove any lipid produced by the cells. All experiments were repeated in triplicate using primary adipocyte cells collected from different mice.

### Collection of conditioned media

As the cell cultures were expanded in the second week of growth, media was changed every two days. Media removed from the cells was centrifuged at 700×*g* for 7 min to remove any cells or debris. For adipocyte media, lipid present in the media was removed and discarded. All media was stored at 4 °C and used within a week of collection.

### Cell culture irradiation

Cell cultures were irradiated using a Rad Source RS-2000 X-ray box irradiator. Low energy X-rays were attenuated with a copper filter. A constant dose rate of 2 Gy/min was used in every experiment regardless of the total dose delivered. Dosimetry is performed by UNMC Radiation Safety Office regularly to verify the uniformity of dose in the center and at three points along each of the edges of the radiation field.

### Cell viability

Cells were seeded at 1 × 10^5^ cells/well in 6 well plates for 24 h and then irradiated with 5 Gy of X-rays. After 5 days, the media was collected, cells trypsinized, and the mixture was pelleted by centrifugation at 700×*g* for 5 min. Cells were resuspended in 200 µL of fresh media, a small aliquot was mixed 1:1 with trypan blue and living or dead cells were enumerated using a hemocytometer. For experiments where adipocytes were combined with fibroblasts, 5 × 10^4^ cells of each cell type were mixed and seeded together in the same well. For co-culture experiments, a transwell insert was seeded with 1 × 10^5^ cells, the transwell was removed at the time of irradiation and then returned to the well immediately after irradiation. For conditioned media experiments, cells were seeded in normal growth media one hour prior to radiation exposure and the media was replaced with conditioned media from either adipocytes or fibroblasts. Heat inactivated media was incubated at 60 °C for one hour and cooled to room temperature prior to its addition to fibroblast cell cultures.

### Size exclusion columns for protein separation in cell culture media

Media from adipocytes were collected after 48 h of culture. Fifteen mL of media was applied to Amicon Ultra-15 centrifugal filter units (Millipore, cat. C7715, Darmstadt, Germany). The 3 kDa filter unit was centrifuged at 4,000×g, 25 °C, for 40 min. The 50 kDa filter unit was centrifuged at 4,000×g, 25 °C, for 15 min. The ultrafiltrates (< 3 kDa and < 50 kDa) and retained concentrates (> 3 kDa and > 50 kDa) were collected then sterile filtered through a 0.2 µm syringe filter. The concentrates are retained in a volume of 200–300 µL, so these were brought up to the required volume with normal growth media. As a control, unseparated media from adipocytes were also collected and sterile filtered. The filtered medias were applied to cultured fibroblasts one hour prior to 5 Gy of irradiation and 5 days later, the cells were assayed for viability.

### Adiponectin ELISA

Cell culture media was assayed to determine the concentration of adiponectin secreted by either fibroblasts or adipocytes. A mouse adiponectin ELISA kit (Abcam, cat. 108785) was used according to manufacturer’s protocol. Briefly, 1 mL of media was collected from cells after 48 h in culture and centrifuged at 3,000×g for 10 min at 4 °C to remove any debris. Duplicate wells were incubated with 100 µL of media or provided standards for 3 h at room temperature. Wells were washed 5 times then incubated with biotin conjugated adiponectin antibody for 1 h, followed by another 5 washes. A streptavidin conjugated secondary was added for colorimetric detection of adiponectin bound to the plate. The absorbance was read at 450 nm and 570 nm (for background correction), the background subtracted absorbances were used to determine a standard curve and calculate the adiponectin concentrations in media samples.

### Collagen contraction

The collagen contraction assay was performed as previously described^13,35^. Fibroblasts were seeded in T25 flasks at 5 × 10^5^ cells per flask with 5 mL of normal growth media. Twenty four hours after seeding, the cells were either sham irradiated or given 2 Gy of X-rays. Cells were harvested 48 h after treatment and resuspended at 4.5 × 10^5^ cells per 100 μL of FBS. Rat tail collagen (Corning, cat. 354249, Corning, NY, USA) was diluted in 0.5 M glacial acetic acid and 10X DMEM to a final concentration of 2 mg/mL. Cells were embedded in the collagen at 1.5 × 10^5^ cells per 500 μL in a low attachment 24-well plate. Once the collagen discs were solidified, the collagen was released from the sides of the well, and suspended on 500 μL complete growth media. After 5 h of contraction, the discs were imaged and the area of the collagen discs were measured using ImageJ software.

### β-Galactosidase for cellular senescence

We have previously described the senescence staining procedure^8,35^. This protocol was adopted from Eccles and Grace, and optimized for mouse primary fibroblasts^56^. Fibroblasts were seeded in T25 flasks at 2 × 10^5^ cells per flask with 5 mL normal growth media. Twenty four hours after seeding, media was refreshed with either normal growth media ± 0.3 ng/mL recombinant mouse adiponectin, fibroblast conditioned media, or adipocyte conditioned media. After one hour incubation, the cells were either sham irradiated or given 3 Gy of X-rays. At 48 h post-irradiation, the cells were washed and fixed in 4% PFA in PBS for 3 min. The monolayer was washed twice with PBS for 5 min and then covered in SA-β-Gal staining solution (0.1% X-gal, 5 mM potassium ferrocyanide, 5 mM potassium ferricyanide, 150 mM sodium chloride, and 2 mM magnesium chloride in 40 mM citric acid/sodium phosphate solution, pH 6.0). Cells were incubated in a dark, 37 °C incubator for approximately 24 h. Images of the cell cultures were captured with a Leica DMi1 inverted microscope outfitted with a Leica MC170 HD camera. Images were imported into ImageJ and inverted to better visualize and enumerate cells positive (pink) for senescence staining. For each experiment, 8–10 fields from each sample were analyzed.

### Prostate cancer and colorectal cancer cell culture

Cancer cell cultures were maintained as we have previously described^35,57,58^. PC3 cells were obtained from the American Type Culture Collection (ATCC, cat. CRL-1435) and grown in RMPI-1640 media supplemented with 10% FBS and 1% penicillin/streptomycin. HT29 cells were obtained from the ATCC (cat. HTB-38). The cells were grown in DMEM media supplemented with 10% FBS, 1% penicillin/streptomycin and 1% non-essential amino acids. Cells were maintained at 37 °C in the presence of 5% CO_2_. Cells were passaged every 2–3 days for no more than 20 passages. Cells were routinely tested for mycoplasma contamination and were mycoplasma negative.

### Clonogenic survival

The clonogenic survival assay was performed as previously described by us^35,57^. Cancer cell (PC3 and HT29) cultures in log phase of growth were seeded onto 6 well plates and treated overnight with 0.3 ng/mL of recombinant human adiponectin or an equal volume of PBS for control. The following morning, cells were either sham irradiated or treated with 3 Gy of radiation. Following treatment, the cells were trypsinized and counted. Serial dilutions were performed and 500 cells were seeded onto 6-well plates in triplicate. Colonies were allowed to form for 10 days, then fixed and stained with 0.5% Crystal Violet and 5% methanol. Colonies were observed using a dissecting microscope and only colonies containing > 50 cells were counted. The fraction of surviving cells was normalized to the plating efficiency of the sham treated cells.

### Statistical analysis

All statistical analyses were performed with GraphPad Prism software v6.0.5. All graphical representations of data are the mean ± standard deviation of three independent experiments. All primary cell cultures were isolated from different animals to obtain three true biological replicates. Statistical significance was calculated using 1-way ANOVA (alpha = 0.05) followed by a post hoc Tukey’s test for multiple comparisons to determine adjusted *p* values.

## Data Availability

All data generated or analysed during this study are included in this published article.
